# Molecular replacement: tricks and treats

**DOI:** 10.1107/S0907444913015291

**Published:** 2013-10-12

**Authors:** Chantal Abergel

**Affiliations:** a Information Génomique et Structurale, IGS UMR 7256, CNRS, Aix-Marseille Université, IMM, FR3479, 163 Avenue de Luminy – case 934, 13288 Marseille CEDEX 09, France

**Keywords:** molecular replacement

## Abstract

Molecular replacement is the method of choice for X-ray crystallographic structure determination provided that suitable structural homologues are available in the PDB. Presently, there are ∼80 000 structures in the PDB (8074 were deposited in the year 2012 alone), of which ∼70% have been solved by molecular replacement. For successful molecular replacement the model must cover at least 50% of the total structure and the C_α_ r.m.s.d. between the core model and the structure to be solved must be less than 2 Å. Here, an approach originally implemented in the *CaspR* server (http://www.igs.cnrs-mrs.fr/Caspr2/index.cgi) based on homology modelling to search for a molecular-replacement solution is discussed. How the use of as much information as possible from different sources can improve the model(s) is briefly described. The combination of structural information with distantly related sequences is crucial to optimize the multiple alignment that will define the boundaries of the core domains. PDB clusters (sequences with ≥30% identical residues) can also provide information on the eventual changes in conformation and will help to explore the relative orientations assumed by protein subdomains. Normal-mode analysis can also help in generating series of conformational models in the search for a molecular-replacement solution. Of course, finding a correct solution is only the first step and the accuracy of the identified solution is as important as the data quality to proceed through refinement. Here, some possible reasons for failure are discussed and solutions are proposed using a set of successful examples.

## Abstract 2

To be successful, molecular replacement relies on the quality of the model and of the crystallographic data. Some tricks that could be applied to the models or to the crystal to increase the success rate of MR are discussed here.

## 1. Introduction

Since the early days of crystallography, rationalization of protein crystallogenesis, the first step in crystallography, has been the Holy Grail of protein crystallographers. Today, protein crystallization is less of a bottleneck; however, this is mostly owing to progress in high-throughput screening, including protein production, purification and crystallization [expression of tagged proteins (Kim *et al.*, 2011 ▶; Abergel *et al.*, 2003 ▶), commercial screens for crystallization (Slabinski *et al.*, 2007 ▶; Luft *et al.*, 2011 ▶) and robotics and/or optimized experimental design (Audic *et al.*, 1997 ▶)]. Since this does not rely on a theoretical understanding of crystallogenesis, most biologists sense crystallization as ‘magic’. Along the same lines, once usable crystals have been produced, crystallography relies on physics, an exact science, and it should thus be straightforward to proceed from the diffraction data to an electron-density map. In fact, succeeding in getting a protein to crystallize is definitely not the final hurdle in the determination of its three-dimensional structure. Molecular replacement (MR) remains the method of choice for structure determination, especially since the international structural genomics effort in the last decade has provided a tremendous increase in the number of structures deposited in the Protein Data Bank (PDB; http://www.pdb.org; Berman *et al.*, 2000 ▶). Even if molecular replace­ment can sometimes be straightforward, it can also provide incorrect solutions or solutions that are not refinable. Here, we will discuss some of the most evident bottlenecks that occur when there is low sequence identity between the reference structure and the target or when the reference structure only provides a partial solution (multi-domain proteins or structures of complexes). In such cases, the data resolution can be critical to enable the refinement process. We will also discuss the case of flexible macromolecules that can assume different conformations depending on the crystallization conditions. We will propose possible solutions to overcome each of these common problems.

## 2. Low sequence identity between the template and the target

Molecular replacement is the least expensive and the fastest crystallographic method for solving the structure of a protein. However, this approach requires the availability of at least one structural homologue sufficiently close to the target. During the last decade, structural genomics projects have provided hundreds of reference structures to the scientific community, increasing the probability of finding structural homologues in the PDB. At the same time, bioinformatics techniques for detecting low sequence similarity have continued to improve, allowing more distant putative three-dimensional homologues to be identified (Shi *et al.*, 2001 ▶; de Bakker *et al.*, 2001 ▶; Marks *et al.*, 2011 ▶; Cong & Grishin, 2012 ▶; Hopf *et al.*, 2012 ▶). However, the most obvious case of MR failure is when the sequence homology between the reference structure and the target to be solved is low. The first parallel between sequence and structure homology was provided almost 30 years ago by establishing a link between the C_α_ r.m.s.d. between two structures and their percentage sequence identity (Chothia & Lesk, 1986 ▶). In most cases of successful MR, the protein of interest shares at least 35% sequence identity with its structural homologue, which corresponds to a C_α_ r.m.s.d. of around 1.5 Å. Below this threshold, and down to 20% sequence identity, the overall fold is usually well conserved but the differences in the three-dimensional structures become too large to be handled by the standard MR protocol. Although the success rate of MR drops considerably when the sequence identity between the template and target proteins is below 35%, it has been found that screening for MR solutions with a large number of different homology models may still produce a suitable solution when the original template has failed (Turkenburg & Dodson, 1996 ▶).

## 3. The accuracy of the model is essential

**Figure 1:**
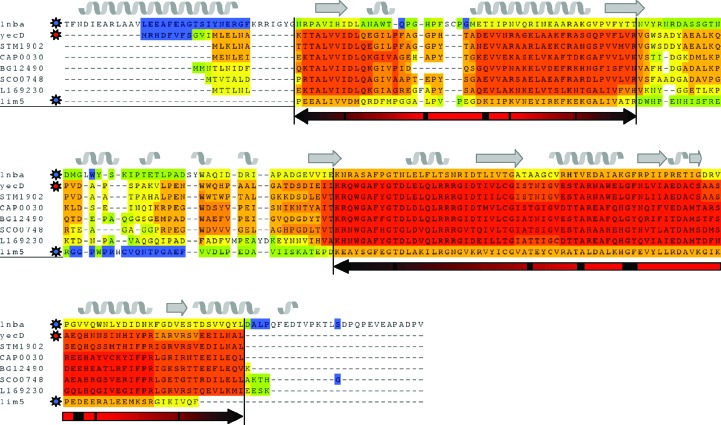
3*D-Coffee* structural alignment of the *E. coli* YecD sequence. The consistency of the alignment is given by the CORE index, with a colour code from blue (inconsistent) to red (highly consistent). Secondary-structure elements have been added at the top of the alignment based on the *Arthrobacter* *N*-­carbamoylsarcosine amidohydrolase structure (PDB entry 1nba; Romao *et al.*, 1992 ▶). The red arrows at the bottom of the alignment correspond to the core domain of the structure.

**Figure 2:**
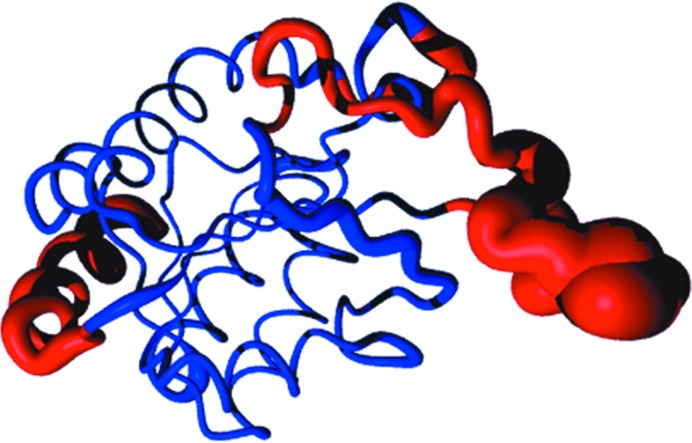
Representation of the models in ‘sausage’ mode. All model structures are superimposed and an average structure is computed. The average C_α_ r.m.s.d. between the mean structure and the models at a given position is illustrated by the diameter of the ribbon in the figure. Deleted regions are represented in red.

This raises the question of how to generate accurate models. One way would be to use as much information as possible to retrieve the features that drive a sequence to fold in a specific structure. Given the number of available sequences and structures in the databases, multiple alignment is the first option to define the boundaries of secondary-structure elements. 3*D-Coffee* or *Expresso* (Notredame, 2010 ▶; Poirot *et al.*, 2004 ▶; Armougom *et al.*, 2006 ▶) are able to retrieve the best structural homologue for each of the sequences included in the alignment, including the target itself, thus allowing the structural information to be mapped onto the multiple alignment. It also provides a CORE index, which is a measure of the accuracy of the alignment for each sequence at every position in the alignment. Consequently, the parts of the reference structures that are accurately aligned to the target sequence become obvious, as well as those which are inaccurate and could eventually be removed to optimize the models (Fig. 1 ▶). This provides the core structure most likely to provide a molecular-replacement solution. A set of models can then be generated by mapping the target sequence onto the best reference structures based on the *Expresso* CORE index. It can thus happen that the final models correspond to chimeric structures, where one reference structure is used to model part of the target sequence and another reference structure will help to build the rest of the model (PDB entry 1j2r; Claude *et al.*, 2004 ▶). We implemented the *CaspR* server (http://www.igs.cnrs-mrs.fr/Caspr2/index.cgi), which is an automated molecular-replacement procedure using a set of standard crystallo­graphic tools together with multiple alignment. The first step of the process is to produce a robust multiple alignment using the *Expresso* software, and the CORE index information is provided to *MODELLER* (Sali & Blundell, 1993 ▶; Fiser & Sali, 2003 ▶) with a random initial perturbation to generate a large number of different models. With properly selected sequences and structures, *MODELLER* will produce a set of accurate models. Fig. 2 ▶ illustrates the structural space sampled by the main chains of the models, with the width of the ribbon being proportional to the variability between the models and with the core structure deviating less than the loops. The *CaspR* suite will truncate unreliable parts of the alignment, thus doubling the number of models (truncated parts are represented in red in Fig. 2 ▶). Molecular replacement using the *AMoRe* software (Navaza, 2001 ▶) is performed for each model in search of a possible solution. The best solutions in terms of correlation and *R*_work_ are submitted to a refinement process using *CNS* (Adams *et al.*, 1997 ▶; Pannu & Read, 1996 ▶; Brünger *et al.*, 1998 ▶) and the *R*_free_/*R*_work_ can be used to rank the solutions.

The user only provides the crystallographic data (symmetry, reflections and number of molecules per asymmetric unit), a FASTA file with the target sequence and a set of reference sequences, and a set of reference structures (1–6 PDB files). In our experience, the optimal reference sequences are those that will provide a continuous gradient of sequence conservation between the target sequence and the reference structures (for a review, see Barton, 2008 ▶). It is thus crucial to carefully select the reference sequences and the reference structures. Much faster and better programs have now been developed by the crystallographic community (McCoy *et al.*, 2007 ▶; Keegan & Winn, 2008 ▶; Adams *et al.*, 2011 ▶; Vagin & Teplyakov, 2010 ▶; Long *et al.*, 2008 ▶); however, few of them use multiple alignment to generate their models even if the pruning of unreliable parts of the structure can sometimes be proposed. The set of models based on multiple alignment generated by *CaspR* can always be retrieved and used to screen for a molecular-replacement solution using any other available software.

**Figure 3:**
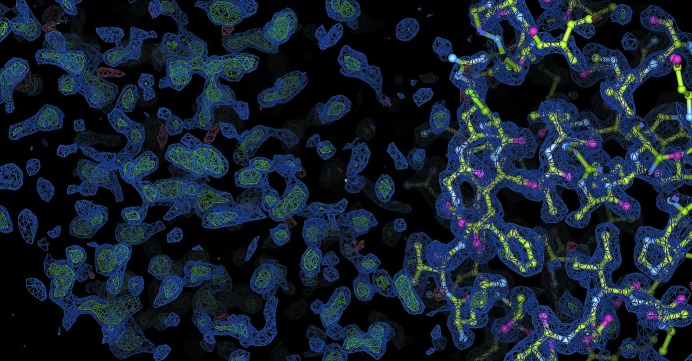
*Coot* image of the molecular-replacement solution (Emsley & Cowtan, 2004 ▶). The lysozyme molecule is shown in ball-and-stick representation. The 2*F*_o_ − *F*_c_ (blue) and *F*_o_ − *F*_c_ electron-density maps (green and red) highlight the quality of the solution around the lysozyme molecule as well as the location of the missing Ivy structure.

**Figure 4:**
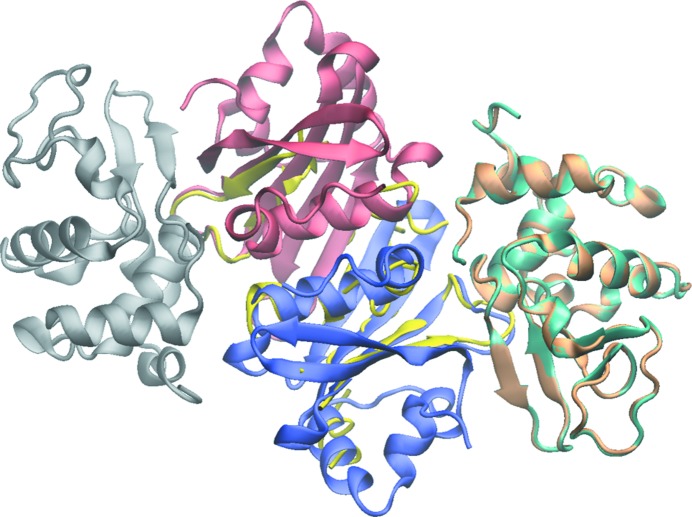
Cartoon representation of the Ivy–lysozyme complex. Lysozyme molecules are coloured grey (first monomer), pale brown (second monomer, initial solution) and light blue (second monomer, final solution). An intermediary stage of the Ivy dimer construction using *BUSTER* for refinement is coloured yellow. The monomers in the final Ivy dimer structure are coloured red and blue. The figure was produced using the *VMD* software (Humphrey *et al.*, 1996 ▶).

It is now worth discussing a problem that arises when the divergence between the target sequence and the reference sequences/structures spans a large fraction of the target sequence. In such cases, the resulting truncations may make the refinement of true molecular-replacement solutions tedious. This can also happen when working with multi-domain structures (or complexes of macromolecules) where there is a structural homologue available for only one domain (or one molecule of the complex). We experienced such a case with an *Escherichia coli* ORFan protein which was serendipitously identified as an inhibitor of vertebrate lysozyme (Ivy; Monchois *et al.*, 2001 ▶). The Ivy protein has the same molecular weight as its substrate and was by chance cocrystallized with the historical protein hen egg-white lysozyme, a reference structure for all crystallographers. Molecular replacement unambiguously provided a clear solution with two molecules of lysozyme per asymmetric unit and sufficient space to place two more equivalent molecular-weight molecules; the Ivy protein was characterized as a dimer in solution (Fig. 3 ▶). At the time we used the *BUSTER* software (Blanc *et al.*, 2004 ▶) to iteratively extract the Ivy structure information, and the refinement process was only made possible owing to the quality of the diffraction data, which were complete in all resolution shells, were highly redundant and, more importantly, extended to 1.6 Å resolution (Fig. 4 ▶; Abergel *et al.*, 2007 ▶). In recent decades, much effort has been invested in refinement and in automatic building programs, and their use would probably have reduced the time and effort that we invested to progressively build the Ivy structure. For example, *Buccaneer* (Cowtan, 2006 ▶) allows the Ivy dimer structure to be built from the phases provided by the molecular-replacement solution obtained with two lysozyme molecules and, given the resolution of the data, *Buccaneer* performed even better when the starting phases were run through *ACORN* (Yao, 2002 ▶). Owing to the expected divergence between the model and the target structure, molecular replacement is usually performed using the low-resolution part of the data. However, once a solution has been identified, the refinement and building steps will become increasingly straightforward when the resolution of the data also increases. A lack of high-resolution data may thus be detrimental to the refinement process. Different methods have been proposed in order to try to improve the diffraction resolution (reviewed in Heras & Martin, 2005 ▶), and the simplest and most spectacular methods rely on desiccation procedures (Heras *et al.*, 2003 ▶; Abergel, 2004 ▶), which can dramatically improve the data resolution from 10 to 2 Å. This simple method is now becoming of general use even on synchrotron beamlines as a systematic manoeuvre to improve resolution and in some cases as the only way to ‘rescue’ hopeless protein crystals.

## 4. Flexible structures

**Figure 5:**
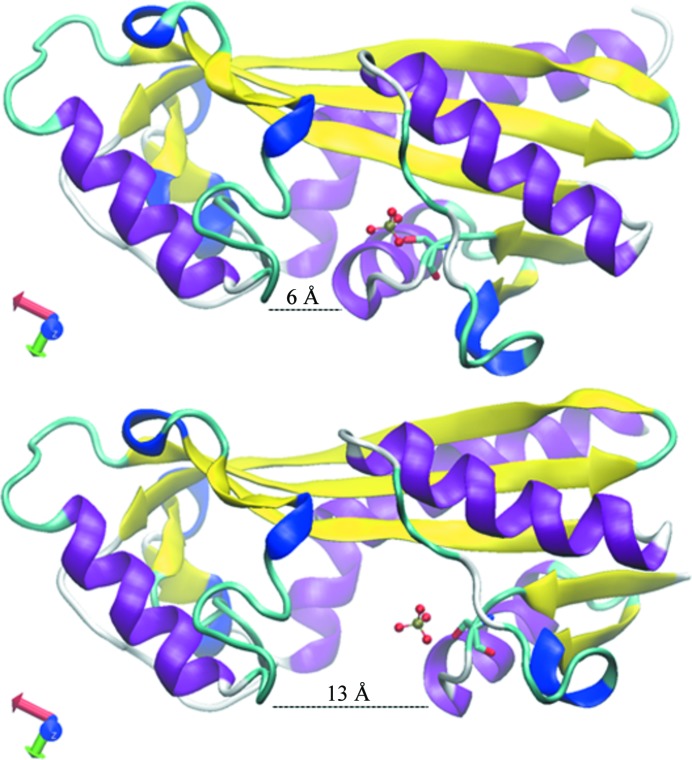
Cartoon representation of the *S. aureus* RdgB protein structure. The phosphate molecule and the serine residue at the active site are shown in ball-and-stick and in stick representation, respectively. The width of the active side owing to the change in conformation is marked for each monomer.

Proteins are biological entities that present various degrees of flexibility reflecting their molecular functions. They can adopt different conformations to perform catalysis, ligand binding and allostery. Extreme flexibility is exemplified by intrinsically disordered proteins that become structurally ordered under specific conditions, while structural proteins present the exact opposite behaviour, with their flexibility being confined to side chains. The vast majority of proteins are between these two extremes. *In vitro*, depending on the crystallization conditions (*i.e.* temperature, ionic strength and pH), proteins will be trapped in the crystal in a given conformation that may not be the same in their homologues in the PDB. In such cases, when the template structure is directly used to interpret the diffraction data, the molecular replacement may fail since only part of the structure will be properly positioned into the asymmetric unit. Even worse, the correct (partial) solution may eventually be rejected owing to clash problems. An alternate possibility would be to separate the different domains of the reference structure and to perform molecular replacement using each domain sequentially to extract the complete solution. Once again, the accuracy of the domain prediction becomes crucial and the multiple alignment must be as accurate as possible in order to properly delineate the domain boundaries and to produce the best possible models for each subdomain before performing the multi-body search. However, when there are many molecules in the asymmetric unit, this approach has the disadvantage of increasing the number of search parameters, diminishing the peak contrast during the first MR steps. This obviously may lead to incomplete solutions that will necessitate iterative steps of refinement and model building, ultimately producing a nonrefinable solution. Some flexible proteins may also be monodomain structures, making it difficult to split the structure to probe possible changes in conformation between the reference structure and the structure that we wish to solve. The same case can arise for oligomeric structures following the sequential model of allosteric regulation (Koshland *et al.*, 1966 ▶), in which different monomers can adopt different conformations in the same crystal. In such cases, subdomain boundaries are also difficult to predict from the protein sequence and alternate computational approaches will have to be used to identify the hinge between the subdomains (see §[Sec sec5]5). The *Staphylococcus aureus* inosine triphosphate pyrophos­phatase is a dimeric enzyme that hydrolyses noncanonical nucleoside triphosphates to prevent their incorporation into DNA and RNA. We obtained a crystal form of this enzyme in which the asymmetric unit contained a dimer consisting of one monomer in an open state and the other in a closed conformation upon binding of a phosphate molecule at the active site of the protein. As a consequence, the active-site pocket underwent a 7 Å closure to give an overall 2.1 Å C_α_ r.m.s.d. between the two monomers (Fig. 5 ▶; PDB entry 4bnq). This is an extreme case where molecular replacement can fail owing to a major change in the conformation of an identical protein sequence inside the same crystal. For instance, the *E. coli* liganded dimer structure could not be solved by molecular replacement using the apo RdgB structure and was solved using the SAD method (Savchenko *et al.*, 2007 ▶). Because we usually assume that there is only one overall conformation in a crystal, it could have been useful to compute the self-rotation in order to evaluate to what extent the conformations of the two monomers were different, with the highest divergence producing pseudo-symmetry peaks with lower correlation. For this peculiar case, using data between 15 and 4 Å resolution, the self-rotation produced a pseudo-symmetry peak with a 37.5% correlation (Winn *et al.*, 2011 ▶).

## 5. Combination of methods

A better approach to solve the structure of flexible proteins (monodomain or multidomain) would be to generate models sampling all of the possible changes in conformation and use them sequentially in a search for a possible molecular-replacement solution. Normal-mode analysis is a very powerful approach to predict macromolecule movements, which in a majority of cases can be modelled by using at most two low-frequency normal modes (Suhre & Sanejouand, 2004*a* ▶). The *elNémo* server, based on the elastic network model (Suhre & Sanejouand, 2004*b* ▶), can be used to compute low-frequency normal modes of macromolecules in order to generate a large number of different conformational models corresponding to different amplitudes of the calculated lowest-frequency normal mode. The set of models can then be used to search for a molecular-replacement solution using the *R*_free_ and *R*_work_ as a hint to the conformational models that are most likely to produce a solution by molecular replacement. The best models can then be further optimized by refining the normal-mode analysis parameters. More evidently, the server can also compute the normal modes that best describe the change in the conformation of a given macromolecule when there are homologous structures in the PDB with different conformations (*i.e.* apo *versus* holo forms) and they do not have to be identical in sequence. The server can then generate intermediate conformational models that can be used in molecular replacement. This approach should be considered as a necessary approach for successful molecular replacement, especially for multi-domain proteins, and normal-mode analysis has now been implemented as a parameter in other molecular-replacement programs such as *Phaser* (McCoy *et al.*, 2007 ▶).

Complex structures are often studied using different approaches combining crystallography, electron microscopy (EM) or small-angle X-ray scattering (SAXS), and a variety of models can be built by combining the information derived from each technique. When working with multimeric structures (oligomers or heteromultimers), the availability of accurate models with their subunits/domains correctly oriented is obviously an advantage during a molecular-replacement search. For oligomeric structures, crystallographic data can provide noncrystallographic symmetry (NCS) information which can be used to retrieve the relative orientation of monomers to build the corresponding oligomer. EM and SAXS data can also provide valuable information on the relative positions and orientations of each molecule (or domain) in oligomers and heteromultimers. It is thus possible to take advantage of the multimerization state of a molecule both in solution and in the crystal by combining the information from X-ray data (NCS) with EM data or SAXS data. For instance, even negative-stained electron-microscopy reconstructions can produce suitable medium-sized protein models for molecular replacement, and the *AMoRe* software (Navaza, 2001 ▶) can use directly an EM reconstruction map to search for an MR solution (Trapani *et al.*, 2006 ▶, 2010 ▶). For successful refinement, the completeness of the X-ray data at low resolution is crucial to provide sufficient overlap between X-ray and EM data. However, as discussed previously, the problem can be complicated when the monomers in oligomeric structures adopt different conformations. In such cases, the most promising strategy is one in which the information from NCS, NMA, EM and/or SAXS is used to generate the oligomeric models. The height of the correlations of the self-rotation peaks corresponding to the NCS provides a hint to the conformational variability of the monomers (the correlation values being inversely proportional to the amplitude of the conformational differences). In such cases, normal-mode analysis can help to find the best alternative conformations to interpret the EM reconstruction by fitting the atomic NMA models in the EM map. In return, the best-fitted EM reconstruction can be used to construct the best oligomeric atomic model which is of high resolution compared with the EM map. This approach can also be used to filter putative MR solutions by comparison with the EM fitted model. For macromolecular complexes, this approach can also be used when there are structural homologues for each molecule of the complex available in the PDB, but the presence of multiple copies of the same molecule will provide the NCS necessary for successful refinement (Trapani *et al.*, 2010 ▶).

## 6. Concluding remarks

During the past decades, the structural biology community has invested a great effort in bioinformatics and structure-prediction optimization. One example is the worldwide CASP (Critical Assessment of protein Structure Prediction) competition, which is devoted to protein structure prediction and has taken place every two years since 1994 (Moult *et al.*, 1995 ▶). Promising approaches using global statistical methods have recently emerged using maximum entropy (Marks *et al.*, 2011 ▶; Morcos *et al.*, 2011 ▶; Hopf *et al.*, 2012 ▶), Bayesian networks (Burger & van Nimwegen, 2010 ▶) or covariance estimation (Jones *et al.*, 2012 ▶; Meinshausen & Bühlmann, 2006 ▶). For instance, the *EVfold* software uses the maximum-entropy approach to infer evolutionary co-variations between sequences belonging to the same structural family, an approach that was anticipated a long time ago (Göbel *et al.*, 1994 ▶; Shindyalov *et al.*, 1994 ▶) and that now seems to produce very promising results. Briefly, a set of constraints is extracted from a multiple alignment through the analysis of residue correlations. The inferred residue-pair couplings provide the information on the residue three-dimensional proximity, which is used to compute the fold of the protein structure (Marks *et al.*, 2011 ▶). This approach has been demonstrated successfully for some membrane proteins (Hopf *et al.*, 2012 ▶) as well as for a set of 50- to 260-amino-acid proteins (Marks *et al.*, 2011 ▶). For the correctly predicted structures the error (C_α_ r.m.s.d. > 2.7 Å) would probably not directly provide useful models for molecular replacement. However, no structural information was used to compute the multiple alignment and the possible flexibility of the proteins was also not taken into account. It is thus likely that these models could be optimized by adding structural information from other experimental (*i.e.* EM/SAXS) and/or theoretical (NMA) sources. Since the models were built *ab initio*, any improved model is worth trying in molecular replacement when diffraction data are available as it may lead to clear solutions. It thus appears that some light can finally be seen at the end of the tunnel regarding protein structure prediction, especially when combining these predictions with experimental data. As the icing on the cake, this approach could increase the success rate of molecular replacement even for new structural families.
